# Ferroptosis as a Novel Therapeutic Strategy to Overcome Multidrug Resistance in Colorectal Cancer

**DOI:** 10.3390/ph19020252

**Published:** 2026-02-01

**Authors:** Dina Mahemuti, Lanfei Ma, Waqas Siddiqe, Ziyue Tang, Yuxin Kong, Wenfang Li, Zhiwei Zhang, Zhengding Su, Ayitila Maimaitijiang

**Affiliations:** 1 School of Pharmaceutical Science, Institute of Materia Medica, College of Life Science and Technology, Xinjiang University, Urumqi 830017, China; dina333@stu.xju.edu.cn (D.M.); 107552403479@stu.xju.edu.cn (L.M.); waqassiddiqe23@gmail.com (W.S.); tangziyue@stu.xju.edu.cn (Z.T.); liwenfang@xju.edu.cn (W.L.); zhangzzw@xju.edu.cn (Z.Z.); 2 College of Smart Agriculture, Xinjiang University, Urumqi 830046, China; kongyuxin@stu.xju.edu.cn

**Keywords:** ferroptosis, colon cancer, chemotherapy, drug resistance

## Abstract

Colon cancer (CC) remains a leading cause of cancer-related mortality worldwide, with multidrug resistance (MDR) presenting a formidable barrier to successful chemotherapy. Ferroptosis—an iron-dependent, lipid peroxidation-driven form of cell death—offers a novel therapeutic avenue to bypass MDR by exploiting metabolic vulnerabilities distinct from traditional apoptosis pathways. Emerging evidence reveals a dynamic interplay between MDR and ferroptosis: MDR cancer cells suppress ferroptosis through *NRF2*/*GPX4*-mediated antioxidant upregulation, iron sequestration by ferritin, and lipid metabolism reprogramming, including *SREBP1*-driven monounsaturated fatty acid accumulation, while ABC transporters actively efflux ferroptosis inducers. On the other hand, ferroptosis inducers such as erastin and RSL3 have the potential to overcome apoptotic resistance and avoid efflux pathways, which recover therapeutic efficacy. This review first describes the primary mechanisms of chemotherapy resistance in colon cancer and then explains the molecular processes that prevent ferroptosis in resistant cells. We also review recent data on the complex interactions between resistance to chemotherapy and ferroptosis, and outline approaches that may stimulate iron accumulation to reverse MDR. By emphasizing novel methods to induce ferroptosis, this review highlights that this approach is a promising strategy to overcome chemotherapy resistance in colon cancer and will facilitate the development of more precise and efficient treatment.

## 1. Introduction

**Figure 1:**
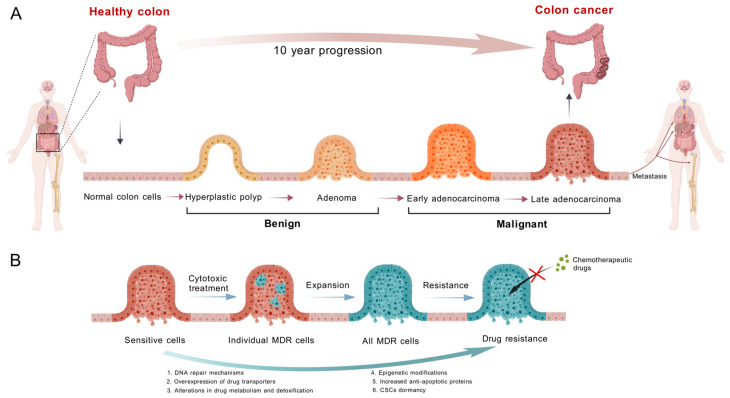
The development process of colon cancer. (**A**) Different stages of colon cancer. (**B**) The process of drug resistance development in colon cancer.

Colon cancer is a malignant tumor of the gastrointestinal system and ranks as the third most common cancer globally, accounting for approximately 10% of all cancer cases. In 2020, there were over 1.9 million new cases of colon cancer and more than 930,000 colon cancer-related deaths worldwide. It is estimated that by 2040, the incidence of colon cancer will increase to 3.2 million new cases per year (a 63% increase), and the mortality rate will rise to 1.6 million deaths per year (a 73% increase) [1]. The incidence is generally higher in males than in females, and there is a trend towards diagnosis in younger populations [2]. Chemotherapy, radiotherapy, and immunotherapy are the primary treatment modalities for cancer patients. However, nearly all chemotherapeutic agents and immunotherapeutic drugs develop drug resistance in patients over time, leading to reduced cytotoxic efficacy against cancer cells. The onset of multidrug resistance (MDR) during postoperative chemotherapy markedly reduces clinical treatment outcomes in colon cancer (Figure 1). MDR is a major reason why chemotherapy does not work as well and why colon cancer patients do not live as long. To improve the clinical management of colon cancer and increase patient survival rates, it is important to reverse MDR in colon cancer cells so that they can respond to chemotherapy again [3].

Ferroptosis, a novel type of cell death first proposed by Dixon in 2012, is a unique, iron-dependent mechanism of regulated cell death induced by the excessive accumulation of lipid peroxides, fundamentally differing from conventional cell death pathways such as apoptosis, necrosis, and autophagy [4]. The discovery of ferroptosis has established a novel conceptual framework for the treatment of various diseases. The development and progression of MDR in colon cancer and ferroptosis are linked in a complicated way that involves interactions between metabolic pathways, genetic mutations, and the tumor microenvironment. Research indicates that the induction of ferroptosis can impede the proliferation of colon cancer and counteract chemoresistance [5,6,7]. Numerous chemotherapeutic agents have been recognized as inducers of ferroptosis, indicating that the modulation of ferroptosis may offer an innovative approach to overcoming multidrug resistance (MDR) in colon cancer.

While existing studies have separately explored the mechanisms of ferroptosis induction or the pathways of MDR formation, there is still a lack of systematic reviews on their dynamic interactive network in colon cancer, particularly as their synergistic therapeutic potential has not been sufficiently explored. This review systematically elucidates the mutual regulatory network between MDR and ferroptosis in colon cancer, clarifying the underlying molecular mechanisms, metabolic reprogramming, and microenvironment-driven synergistic effects. It focuses particularly on how a dual-targeting strategy—simultaneously inhibiting MDR-related pathways and activating ferroptosis—can achieve more efficient and durable antitumor outcomes. We hope this article will provide a new theoretical framework and translational research directions for overcoming chemotherapy resistance in colon cancer.

## 2. Colon Cancer Multi-Drug Resistance

### 2.1. Definition and Clinical Significance of MDR

Multidrug resistance (MDR), a multifactorial phenomenon first described by Biedler and Riehm in 1970, is the complex process by which tumor cells develop cross-resistance to a broad spectrum of drugs with diverse structures and distinct mechanisms of action. It can be classified into two types: primary resistance (preexisting before therapy) and acquired resistance (induced following treatment) [8]. Specifically, cancer cells are considered to exhibit the MDR phenotype when they develop resistance to structurally and functionally unrelated drugs, including agents to which they have not been previously exposed [9]. Once MDR occurs, the anticancer efficacy of chemotherapeutic drugs is significantly diminished. Therefore, MDR represents a primary cause of chemotherapy failure in cancer and is closely associated with tumor metastasis and recurrence. Effectively addressing MDR necessitates an urgent need to elucidate the underlying mechanisms of drug resistance and identify relevant therapeutic targets.

### 2.2. Mechanisms of MDR in Colon Cancer

There are three main types of pathways that can lead to MDR: drug-dependent MDR, target-dependent MDR, and drug-target-independent MDR. Drug-dependent MDR happens when there are too many enzymes that help the body get rid of drugs and transporters that help drugs leave cells. These enzymes and transporters speed up drug metabolism or slow down drug accumulation inside cells. Target-dependent MDR occurs when the drug target undergoes modifications, such as translocation, deletion, mutation, or gene amplification, leading to structural or functional anomalies that diminish drug binding efficiency; for instance, *EGFR* mutations can induce resistance to tyrosine kinase inhibitors [10]. In contrast, drug-target-independent multidrug resistance (MDR) arises from genetic or epigenetic modifications in cellular signaling pathways that desensitize downstream effects of the target, thereby reducing the drug’s efficacy [11].

#### 2.2.1. Overexpression of Drug Transporters

ATP-binding cassette (ABC) transporters are a superfamily of transmembrane proteins that are found in all living things. They use energy from ATP hydrolysis to move different substrates. They are crucial for maintaining cellular balance, absorbing nutrients, removing toxins, and metabolizing drugs. But their overexpression in colon cancer cells is a big reason why MDR happens. Two transmembrane domains (TMDs) and two nucleotide-binding domains (NBDs) make up most ABC transporters. The TMDs make up the substrate translocation channel, which recognizes and binds to specific substrates. The NBDs, on the other hand, are very stable ATP-binding sites that change shape when ATP binds and breaks down, which makes it easier for substrates to cross the membrane [12]. This process adheres to the “Alternating Access Model,” whereby ATP binding induces conformational changes in the TMDs, exposing the substrate from an inward-facing binding site to the extracellular space for release [13].

The human genome encodes 48 ABC transporters, classified into seven subfamilies (A-G) with distinct functions (Table 1) [14]. Among these, P-glycoprotein (P-gp), Multidrug resistance-associated protein 1 (MRP1), and Breast cancer resistance protein (BCRP) are critically implicated in the development of resistance to chemotherapeutic drugs.

**Figure 2:**
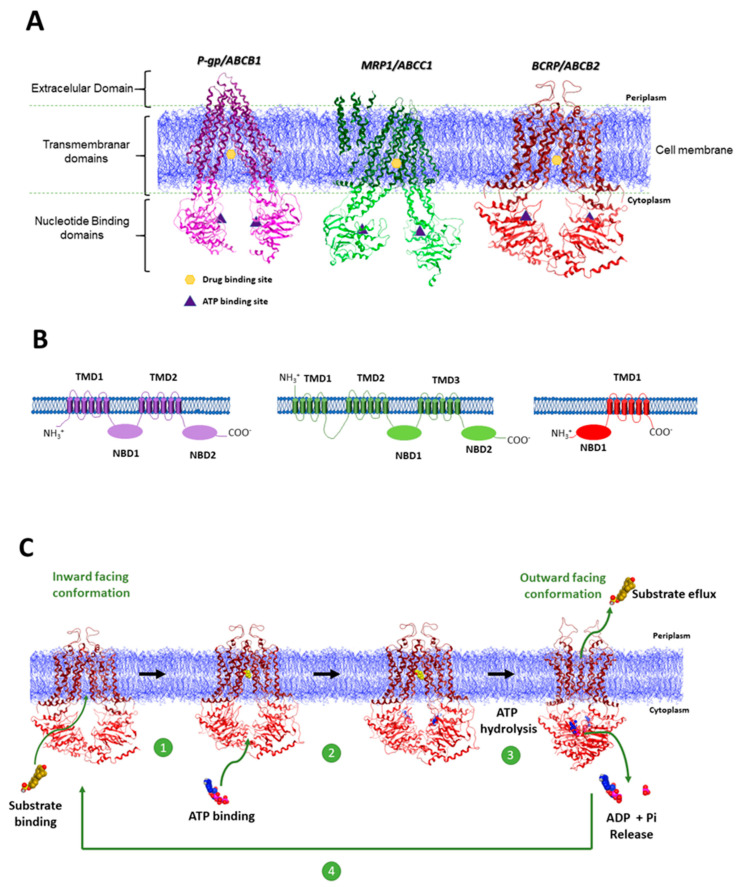
(**A**) High-resolution 3D structure of the mouse P-gp (PDB-ID:5KPI), which has 87% sequence identity to human P-gp; bovine MRP1 (PDB-ID:5UJ9), which has 91% protein identity with the human protein; and human BCRP (PDB-ID:5NJ3). (**B**) Membrane topology models of P-gp, MRP1, and BCRP proteins. (**C**) Simplified representation of the ABC drug efflux mechanism using the 3D structure of BCRP [37].

**Figure 3:**
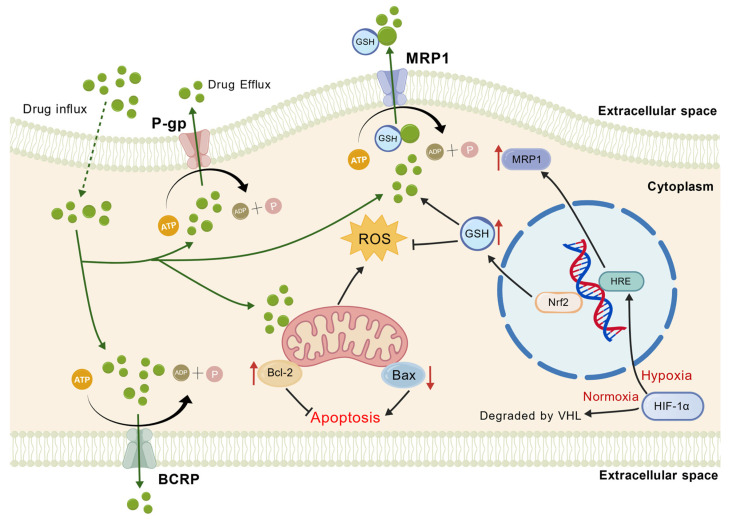
The mechanism of three key ABC transporters (*ABCB1*/P-gp, *ABCC1*/MRP1, *ABCG2*/BCRP) in mediating MDR. MDR in cancer cells is primarily driven by the overexpression of ABC transporters, which efflux chemotherapeutic drugs from the cell, reducing intracellular concentrations and efficacy. This figure depicts P-gp, MRP1, and BCRP embedded in the plasma membrane. Utilizing energy from ATP hydrolysis, these transporters actively pump a wide range of structurally unrelated anticancer drugs from the cytoplasm or membrane leaflets across the plasma membrane into the extracellular space. This efflux mechanism significantly depletes intracellular drug levels, leading to treatment failure.

*ABCB1*, also known as P-glycoprotein (P-gp), is the most extensively studied protein in this context (Figure 2). Comprising 1280 amino acids, it is a transmembrane glycoprotein with a molecular weight of approximately 170 kDa [23]. P-gp is highly expressed in the intestine, blood–brain barrier, liver, and kidney tissues. Under normal physiological conditions, P-gp acts as an active efflux transporter, pumping a wide range of endogenous and exogenous substances out of cells, preventing intracellular accumulation and providing physiological protection (Figure 3). However, its overexpression in tumor cells significantly impairs the efficacy of chemotherapy [16,24]. *ABCB1* expression levels are closely associated with the prognosis and treatment response of colon cancer patients. Molecular subtyping studies have classified colon cancer into four consensus molecular subtypes (CMS), with CMS4 (mesenchymal) tumors exhibiting high *ABCB1* expression, which correlates significantly with poorer survival rates and chemotherapy resistance. Thus, *ABCB1* (P-gp) serves as a critical prognostic biomarker in colon cancer, with its overexpression being an independent predictor of unfavorable clinical outcomes. Clinical sample analyses confirm that high *ABCB1* expression, detectable by immunohistochemistry or qRT-PCR, is not only a prognostic indicator but also a potential biomarker for predicting diminished response to oxaliplatin treatment [25]. Recent studies have shown that *ABCB1* activity is not only regulated by gene expression but also closely related to the tumor microenvironment. For example, hypoxia increases the expression of *ABCB1* through the HIF-1α signaling pathway, contributing to the oxaliplatin resistance of colon cancer stem cells (CD133+). Additionally, the broad and flexible substrate-binding site of P-gp allows it to efflux a wide spectrum of chemotherapeutic agents, underpinning its central role in multidrug resistance [26].

MRP1 (Multidrug resistance-associated protein 1) is made up of 1531 amino acids and weighs about 190 kDa. It is encoded by the *ABCC1* gene (Figure 2). It is expressed on the outer surface of the plasma membrane. Widely distributed in human tissues, its primary function is ATP-dependent active efflux of endogenous or exogenous substances from cells, thereby participating in drug metabolism, detoxification, and the development of drug resistance. Unlike P-gp, MRP1 transport relies on the co-transport of glutathione (GSH) and preferentially effluxes hydrophilic anionic compounds (Figure 3) [27]. MRP1 functions through two main mechanisms: Firstly, it directly effluxes chemotherapeutic drugs. MRP1 overexpression leads to a significant reduction in intracellular drug concentration, diminishing cytotoxicity. Silencing *MRP1* expression via RNAi can reverse cisplatin resistance in A549/DDP cells. Secondly, it acts synergistically with GSH. MRP1 effluxes GSH and its oxidized form (GSSG), lowering intracellular GSH levels, affecting drug detoxification and oxidative stress responses, and indirectly promoting resistance [28]. Studies indicate that hypoxia in the colon cancer microenvironment significantly upregulates MRP1 expression through HIF-1α activation of hypoxia response elements (HREs) in the *MRP1* gene promoter, thereby enhancing drug resistance [29]. Animal models confirm that high MRP1 expression reduces colon cancer cell sensitivity to oxaliplatin and 5-fluorouracil (5-FU), while knockdown of *MRP1* expression restores drug sensitivity and induces apoptosis [30].

BCRP (Breast Cancer Resistance Protein, *ABCG2*) is a protein encoded by the *ABCG2* gene. Its monomer has a molecular weight of approximately 72–75 kDa and consists of 655 amino acids. It contains only six transmembrane domains and one ATP-binding site, structurally classified as a “half-transporter” that requires dimerization for full transport activity (Figure 2). BCRP is expressed in tissues such as the placenta, intestine, and mammary glands, participating in physiological processes including placental barrier formation, intestinal absorption, and mammary gland lactation [31]. It was first cloned in 1998 from the multidrug-resistant breast cancer cell line MCF-7/AdrVp and confers tumor resistance by effluxing chemotherapeutic drugs (Figure 3) [22,32].

BCRP shares overlapping substrate specificities with P-gp and MRP1, collectively forming a multidrug resistance network. For example, at the blood–brain barrier, BCRP cooperates with P-gp to restrict the entry of drugs like rosuvastatin into the central nervous system [33]. In breast cancer cells, upregulation of both BCRP and P-gp expression contributes to chemotherapeutic drug resistance [34]. This cooperation allows tumor cells to reduce drug accumulation through multiple mechanisms, thereby enhancing resistance. Simultaneously, their distinct yet complementary functions further amplify resistance. While MRP1 primarily effluxes anionic drugs, BCRP shows a preference for hydrophobic drugs. This functional complementarity significantly broadens the spectrum of drug resistance in tumor cells [35,36].

#### 2.2.2. Altered Drug Metabolism and Detoxification

Recent research reveals that drug-resistant cells reduce drug toxicity by modulating metabolic enzyme activity, enhancing detoxification systems, and altering oxidative stress responses [24]. Drug-metabolizing enzymes directly impact therapeutic efficacy by modifying the chemical structure or activity of anticancer agents.

Phase I metabolism, the initial stage of drug metabolism, primarily involves oxidation, reduction, and hydrolysis reactions. These reactions introduce or expose polar functional groups (like hydroxyl, carboxyl, thiol, and amino groups) to the drug molecule. This makes it more soluble in water and more polar, which helps it be broken down and excreted from the body [38]. The cytochrome P450 (CYP) superfamily comprises the principal enzymes of Phase I metabolism; variations in their activity among individuals directly affect drug efficacy and toxicity [39]. For example, CYP3A4, which is expressed at high levels in the liver and intestines, metabolizes many chemotherapeutic drugs, lowering their concentrations inside cells [40]. CYP2C8 is another enzyme that hydroxylates paclitaxel, which makes it less able to bind to microtubules and makes it more resistant [41]. Phase I metabolism is essential for both activating and deactivating drugs. Some drugs make more toxic metabolites during this phase.

In phase II metabolism, drugs are conjugated with molecules already present in the body (such as glucuronic acid, sulfate, acetyl groups, and glutathione) to form more stable, water-soluble compounds. This makes it easier for the body to get rid of them through urine or feces. Specific enzymes, such as Glutathione S-transferases (GSTs), UDP-glucuronosyltransferases (UGTs), Sulfotransferases (SULTs), and N-acetyltransferases (NATs), are involved in phase II metabolism [42]. The interplay between Phase II metabolism and MDR chiefly pertains to the bifunctional roles of GSTs. GSTs serve dual roles: they act as Phase II enzymes to accelerate the biotransformation and efflux of antitumor drugs, leading to chemoresistance, and they also function as natural regulators of essential key kinases in the mitogen-activated protein kinase (MAPK) pathway, inhibiting their activation and thereby blocking the MAPK pathway, stopping their activation and then stopping apoptotic pathways [43]. Additionally, P-gp expression may be regulated by the pregnane X receptor (PXR), which also controls the expression of UGTs and SULTs. This co-regulation potentially establishes a “metabolizing enzyme-efflux pump” network, enhancing overall drug resistance [44].

The simultaneous expression of CYP3A4 and P-glycoprotein in the liver and intestine creates a synergistic “absorption-metabolism-efflux” barrier. CYP3A4 breaks down foreign substances, and P-gp pumps the byproducts out of cells, which lowers the amount of drugs that can be absorbed. A lot of drugs are substrates for both CYP3A4 and P-gp. This substrate overlap is an important aspect for interaction, which can either speed up or slow down drug metabolism and absorption. For instance, some drugs may be metabolized simultaneously by CYP3A4 and expelled by P-gp, thereby restricting their bioavailability. This mechanism is especially important in the intestines, where P-gp prevents drugs from entering the bloodstream and CYP3A4 metabolizes drugs to make them less harmful or less active [45].

The GSH system is one of the most important ways in which cells get rid of toxins. It lowers toxicity and helps the body get rid of drugs by either directly neutralizing electrophilic compounds or combining with drug metabolites. Aberrant activation of this system is closely associated with tumor multidrug resistance (MDR). GSH synthesis relies on γ-glutamylcysteine synthetase (γ-GCS) and glutathione synthetase (GS), whereas glutathione reductase (GSR) converts oxidized glutathione (GSSG) back to its active form, GSH, preserving the intracellular reducing environment. Higher levels of GSH in tumor cells make them more resistant to chemotherapy drugs. For example, platinum-based drugs are toxic because they lower levels of GSH, but adding more GSH makes them less effective by neutralizing reactive oxygen species (ROS) that drugs create or by directly binding to drug molecules [46]. The GST family is very important for GSH-dependent detoxification. It helps form water-soluble conjugates by forming a thioether bond between GSH and electrophilic drugs, which are then exported from the cell via MRP1/ABCC transporters. Some GST isoenzymes can also directly bind to chemotherapy drugs, preventing them from interacting with DNA targets, thereby creating a physical barrier [47].

The buildup of ROS inside cells is a significant way that chemotherapeutic drugs cause DNA damage and cell death. On the other hand, stimulating the antioxidant enzyme system terminates this process, which is a significant MDR mechanism. In order to minimize the harmful effects of ROS, tumor cells typically increase the activity of their antioxidant enzymes. Antioxidant enzymes, such as the transcription factor Nrf2, superoxide dismutase (SOD), and glutathione peroxidase (GPx), scavenge reactive oxygen species (ROS) and maintain redox homeostasis, protecting cells from ROS-induced DNA damage and apoptosis. But this antioxidant defense can also make chemotherapeutic drugs less effective at killing cancer cells, which can lead to chemoresistance [48].

#### 2.2.3. DNA Repair Mechanisms

There is a complex yet close relationship between DNA repair mechanisms and cancer MDR. Cancer cells avoid DNA damage caused by chemotherapy by improving or changing their ability to repair DNA, constituting a key mechanism underlying MDR.

The Mismatch repair (MMR) system detects errors in DNA replication or mispaired bases caused by chemotherapy, which can lead to apoptosis or repair. MLH1 and MSH2 are important proteins in the MMR system; they form heterodimers (MSH2/MSH6 or MSH2/MSH3) to participate in the repair process. When MMR is missing, cells cannot sense DNA damage, which allows them to avoid apoptosis, live, and eventually become resistant to drugs. Mutations or deletions in the *MLH1* or *MSH2* genes impair MMR, leading to microsatellite instability (MSI). This state raises the rate of genomic mutations and makes tumor cells less responsive to chemotherapy drugs. 5-FU specifically integrates into DNA and makes a ternary complex (TS-TDP) that stops thymidylate synthase from working, which damages DNA. Studies indicate that defects in the MMR system hinder the repair of DNA damage induced by 5-FU. Cells deficient in *MLH1* or *MSH2* are incapable of effectively repairing DNA strand breaks and mismatches induced by 5-FU, facilitating their survival and the emergence of resistance [49,50,51].

Base excision repair (BER) is a vital mechanism for maintaining genome integrity, mainly fixing DNA damage caused by oxidation, alkylation, and deamination. APE1 and XRCC1 are essential pathway proteins that work collectively to fix damage. Oxaliplatin causes DNA crosslink damage, which creates apurinic/apyrimidinic (AP) sites that are substrates for BER. APE1, the enzyme that starts BER, cleaves AP sites to make single-strand breaks (SSBs). Excessive expression of APE1 accelerates the processing of AP sites. However, if there are not sufficient repair proteins downstream, this can cause SSBs to accumulate and make the genome unstable [52]. XRCC1 is involved in both base excision repair (BER) and single-strand break repair (SSBR) pathways, and it can repair DNA damage caused by oxaliplatin. So, when *XRCC1* is overexpressed, SSBR works more efficiently, DNA damage accumulates less, and cells become less sensitive to oxaliplatin. [53,54].

Homologous recombination repair (HRR) is a vital mechanism for repairing DNA double-strand breaks (DSBs) and depends on the normal expression of genes such as *BRCA1/2*. Mutations in *BRCA1/2* or related genes cause homologous recombination deficiency (HRD), rendering cells more sensitive to poly (ADP-ribose) polymerase (PARP) inhibitors [55]. Specifically, PARP inhibitors block the repair of single-strand breaks by inhibiting PARP activity, thereby inducing synthetic lethality in HRR-deficient tumor cells. This mechanism sensitizes tumor cells carrying *BRCA1/2* mutations to PARP inhibitors [56]. However, tumor cells may sometimes restore HRR function through somatic mutations or other mechanisms, leading to acquired resistance to PARP inhibitors [57]. For example, wild-type tumors can upregulate HRR-related proteins, enhancing DSB repair capacity and counteracting the toxicity of PARP inhibitors [58].

#### 2.2.4. Epigenetic Modifications

Epigenetic modifications, including DNA methylation, histone modifications, and non-coding RNA (ncRNA) regulation, modulate gene expression and influence the development of MDR.

DNA methylation, primarily occurring on cytosine within CpG islands and catalyzed by DNA methyltransferases (DNMTs), is closely associated with gene silencing when located in promoter regions. However, gene body methylation may have distinct regulatory functions. Overexpression of DNMTs in colon cancer cells leads to hypermethylation of promoter regions of critical genes. For instance, significantly elevated methylation of the promoter CpG island of the Csk homologous kinase (CHK) gene suppresses its mRNA and protein expression, promoting tumor proliferation, invasion, and drug resistance. Similarly, aberrant methylation status of prostaglandin-endoperoxide synthase 2 (PTGS2) may enhance resistance through epigenetic silencing [59]. *hMLH1*, a core gene of the MMR system, undergoes promoter hypermethylation leading to transcriptional silencing. This causes MMR functional deficiency, resulting in MSI characterized by accumulated mutations in short repetitive sequences within the genome, thereby reducing tumor cell sensitivity to DNA-damaging drugs [60]. *hMLH1* hypermethylation is the primary cause of high-frequency MSI in approximately 45% of sporadic colorectal cancers (CRC) and is closely associated with proximal colon cancer development [61].

Histone modifications encompass a series of chemical alterations on histone molecules, such as acetylation, methylation, phosphorylation, and ubiquitination. These changes regulate MDR through altering the accessibility of chromatin and the activity of gene transcription. Histone modifications dynamically change chromatin structure and function, thereby affecting gene expression. Histone modifications affect drug metabolism and resistance mechanisms in colon cancer cells via various pathways. Firstly, epigenetic modifiers such as histone deacetylases (HDACs) and histone methyltransferases (HMTs) are essential in both tumorigenesis and resistance. HDACs have two important effects on colon cancer MDR: they alter the shape of chromatin and the way genes are expressed. On one hand, HDACs remove acetyl groups from histones, promoting a condensed chromatin structure that reduces transcription factor access to DNA. High expression of HDAC1 and HDAC2 in colon cancer tissues correlates strongly with malignant phenotypes. They silence tumor suppressor genes (e.g., *p53*) via deacetylation, thereby promoting tumor cell proliferation and inhibiting apoptosis and cell cycle arrest. HDAC1/2-mediated deacetylation of histones H3/H4 leads to chromatin compaction at the p21 promoter, inhibiting its transcription. Silencing of *p21*, a cell cycle inhibitor, promotes tumor cell proliferation and evasion of chemotherapy-induced apoptosis [62]. Furthermore, upregulation of HDAC3 in colon cancer is also associated with tumor cell invasiveness and metastatic potential [63]. On the other hand, HDACs can also promote gene expression in colon cancer. HDAC4 enhances *MDR1* transcription factor (e.g., NF-κB) binding and overexpression by deacetylating histone H3 lysine 9 (H3K9) at the *MDR1* promoter [64]. Finally, histone demethylases maintain the resistant phenotype by remodeling chromatin structure. For example, *KDM5B* promotes the survival of chemoresistant cells by removing the H3K4me3 mark and suppressing the expression of tumor suppressor genes [65].

Non-coding RNAs (ncRNAs) are RNA molecules that do not encode proteins, including miRNAs, lncRNAs, and siRNAs. They are widely distributed in the nucleus, mitochondria, and cytoplasm, playing crucial roles in regulating gene expression. These molecules interact with DNA, mRNA, proteins, and other molecules. They are involved in epigenetic regulation, transcriptional control, splicing regulation, and post-translational modifications. Non-coding RNAs (ncRNAs) affect gene expression and signaling pathways related to multidrug resistance (MDR) through complicated networks of control. miRNAs control resistance by directly targeting enzymes or transporters that degrade drugs. For example, miR-145 makes cells more sensitive to oxaliplatin by inhibiting *ABCC1* expression, whereas miR-21 makes cells more resistant by targeting *PTEN* and activating the *PI3K/AKT* pathway [66,67]. In colon cancer, ncRNAs have become key players in networks, replacing proteins in some cases. They control genes related to metabolism and ribosomal proteins, thereby creating epigenetic signatures linked to resistance [68,69].

The interactions between DNA methylation, histone modifications, and ncRNAs create a dynamic regulatory network that sustains the drug-resistant phenotype. This process not only explains the molecular mechanisms that enable tumors to be resistant to drugs but also provides the foundation for developing novel therapeutic approaches. The mechanisms of multidrug resistance (MDR) in colon cancer are closely associated with epigenetic alterations that regulate gene expression, signaling pathways, and cellular metabolism, which, in turn, influence the development of MDR. Consequently, an in-depth understanding of epigenetic regulatory mechanisms and the development of therapeutic strategies aimed at them are essential for addressing multidrug resistance in colon cancer.

## 3. Current Strategies to Overcome MDR in Colon Cancer

### 3.1. Inhibition of Efflux Pumps Targeting ABC Transporters

Targeting ABC transporter efflux pumps represents a key strategy to enhance intracellular drug accumulation and therapeutic efficacy, particularly in MDR and cancer treatment. To overcome P-gp-mediated MDR, researchers have developed multiple generations of P-gp inhibitors. First-generation inhibitors, including cyclosporin A and verapamil, demonstrated inhibitory activity in vitro but faced limitations in clinical application due to toxicity and pharmacokinetic interactions [70]. Second-generation inhibitors, such as Valspodar (PSC833) and Biricodar (VX710), were structurally optimized derivatives of the first generation. While exhibiting improved inhibitory potency in vitro, they are substrates of drug-metabolizing enzymes, leading to adverse drug–drug interactions and metabolic disturbances [71,72]. Third-generation inhibitors, including Zosuquidar (LY335979), Tariquidar (XR9576), and Elacridar (GF120918), offer higher selectivity and lower toxicity. They bind with high affinity (within the nanomolar range) to the NBDs of P-gp, blocking ATP hydrolysis-driven conformational changes and significantly reversing tumor drug resistance [73,74]. However, some third-generation inhibitors exhibited varying degrees of toxicity in clinical trials [75], leading to the discontinuation of most in clinical development. This prompted the development of fourth-generation inhibitors: natural product inhibitors. As the name suggests, these are primarily plant-derived extracts that not only inhibit P-gp activity to reverse MDR but also possess inherent anticancer properties [76]. Additionally, nanotechnology-based inhibitors have emerged, such as liposomes and polymeric nanoparticles encapsulating chemotherapeutic drugs within nanocarriers to bypass P-gp-mediated efflux [77]; Poly (lactic-co-glycolic acid) (PLGA) nanoparticles capable of loading hydrophobic drugs like paclitaxel, enhancing intratumoral drug concentration via the enhanced permeability and retention (EPR) effect [78]; and P-gp inhibitor co-delivery systems where nanoparticles simultaneously load both the chemotherapeutic drug and the inhibitor, achieving synergistic targeting [79].

### 3.2. Epigenetic Regulation

Epigenetic modifications, including DNA methylation and histone modifications, play a crucial regulatory role in MDR in colon cancer. Unlike irreversible genetic mutations, epigenetic alterations possess a reversible nature, thereby offering a unique therapeutic window for pharmacologically “resetting” the epigenetic state of cells and consequently reversing their drug-resistant phenotype. The restoration of tumor suppressor gene expression and remodeling of chromatin states via DNMT inhibitors or HDAC inhibitors can resensitize resistant cells, which holds significant biological and clinical relevance.

DNA methyltransferase inhibitors (DNMTi) restore gene expression through demethylation, enhancing chemosensitivity. For example, 5-azacitidine covalently binds DNMTi, inhibiting DNA methylation and reactivating silenced tumor suppressor genes [80]. Studies indicate that methylation silencing of *hMLH1* in colon cancer correlates with oxaliplatin resistance, and DNMTi may reverse resistance by restoring *hMLH1* expression [81].

Histone deacetylase inhibitors (HDACi) increase histone acetylation, remodel chromatin structure, and activate anti-tumor pathways. Vorinostat, for instance, inhibits class I/II HDACs, upregulating pro-apoptotic genes like *p21* and *Bax*, while downregulating survival signals such as NF-κB [82]. Preclinical studies suggest that HDACi can overcome resistance by inhibiting the *Wnt*/β-catenin pathway (a key driver in colon cancer), though further validation in trials is needed [83]. Additionally, in colon cancer, miR-21 overexpression promotes 5-FU resistance by suppressing the *PTEN/AKT* pathway, and its inhibitors can restore chemosensitivity [84].

### 3.3. Targeted Therapy

Targeting apoptotic pathways is a key strategy to reverse MDR in colon cancer, primarily by inhibiting anti-apoptotic proteins to restore tumor cell sensitivity to treatment. Venetoclax is a selective Bcl-2 inhibitor. Its mechanism consists of directing the Bcl-2 protein to inhibit its interaction with pro-apoptotic proteins, thereby triggering apoptosis in tumor cells. In colon cancer, the expression of Bcl-2 family proteins is markedly elevated; these proteins are essential for tumor cell survival and chemoresistance. Venetoclax blocks Bcl-2, releasing pro-apoptotic proteins and making the outer mitochondrial membrane permeable (MOMP). This causes cytochrome c to be released, apoptotic pathways to be activated, and, ultimately, tumor cells to die [85,86,87].

The function of cellular FLICE-like inhibitory protein (c-FLIP) in colon cancer is mainly linked to its control of apoptosis and signaling pathways. Studies on c-FLIP suggest that its inhibitors can enhance the efficacy of chemotherapeutic agents by restoring Fas-mediated apoptosis. For example, using siRNA to lower *c-FLIP* expression or c-FLIP inhibitors makes colon cancer cells much more sensitive to chemotherapy drugs. Consequently, c-FLIP is regarded as a crucial therapeutic target in colon cancer, and its inhibitors signify potential anticancer agents [88,89]. In colon cancer models, the combination of TRAIL with c-FLIP inhibitors elevated apoptosis rates in resistant cells by 3–5-fold, while cisplatin pretreatment markedly improved TRAIL efficacy by downregulating c-FLIP and upregulating DR5 [90].

### 3.4. Targeting Resistance-Associated Signaling Pathways

MDR in colon cancer is associated with aberrant activation of multiple signaling pathways; targeting key nodes can reverse resistance. Dysregulation of the *PI3K*/*Akt*/*mTOR* signaling pathway is closely linked to chemoresistance in colon cancer. Inhibitors targeting this pathway have been studied to improve treatment outcomes. For instance, miR-218 targets the *PI3K*/*Akt*/*mTOR* pathway, stopping colon cancer cells from moving and invading, which shows that it could have therapeutic effects [91]. Aberrant activation of the *Wnt*/β-catenin pathway is a fundamental mechanism for preserving colon cancer stemness. The Porcupine inhibitor LGK974 decreases the cancer stem cell (CSC) population and promotes differentiation, thereby increasing sensitivity to chemotherapy [92]. β-catenin degraders effectively reduce chemoresistance in colon cancer cells by inhibiting β-catenin activity [93].

## 4. Ferroptosis in Cancer

### 4.1. Definition and Characteristics of Ferroptosis

Ferroptosis is a type of programmed cell death that depends on iron and differs from other types of cell death, such as apoptosis, necrosis, and autophagy. The accumulation of lipid peroxides propels it and relies on the generation of reactive oxygen species (ROS) [94,95]. This concept was first explicitly defined by Dixon et al. in 2012 [4], although related phenomena were initially observed as early as 2003 through the discovery of the small-molecule compound erastin [96]. Its main mechanisms are problems with cellular iron metabolism, low GSH levels, and a compromised antioxidant defense system.

Ferroptosis differs from other forms of cell death because it induces unique changes in cell and subcellular morphology. During ferroptosis induction, mitochondria experience shrinkage, increased bilayer membrane density, outer membrane rupture, and a decrease or disappearance of cristae, whereas the nucleus retains its typical morphology. There are no typical apoptotic structures (such as apoptotic bodies or chromatin condensation) or autophagic structures (such as autophagosomes) [97]. Even though the plasma membrane does not rupture right away, lipid peroxidation destabilizes it, eventually leading to cell death [98] (Table 2).

### 4.2. Biochemical Mechanisms of Ferroptosis

The biochemical mechanisms of ferroptosis center on the “iron-lipid-antioxidant” triad: iron overload induces lipid peroxidation through the Fenton reaction and lipoxygenase (LOX) activation, whereas the inadequacy of antioxidant systems such as glutathione peroxidase 4 (GPX4) and FSP1 results in unregulated oxidative damage. Transcription factors (such as Nrf2 and *p53*) and metabolic enzymes carefully regulate this process, offering multiple potential targets for disease intervention.

#### 4.2.1. Iron Dysregulation and ROS Generation

Iron dysregulation and reactive oxygen species (ROS) generation are fundamental to ferroptosis. Iron, a crucial trace element, participates in vital cellular functions including oxygen transport, DNA replication, mitochondrial respiration, and signal transduction. But when iron levels are out of balance, either excessive or insufficient, it makes excessive ROS, which causes oxidative stress, lipid peroxidation, and ultimately ferroptosis.

**Figure 4:**
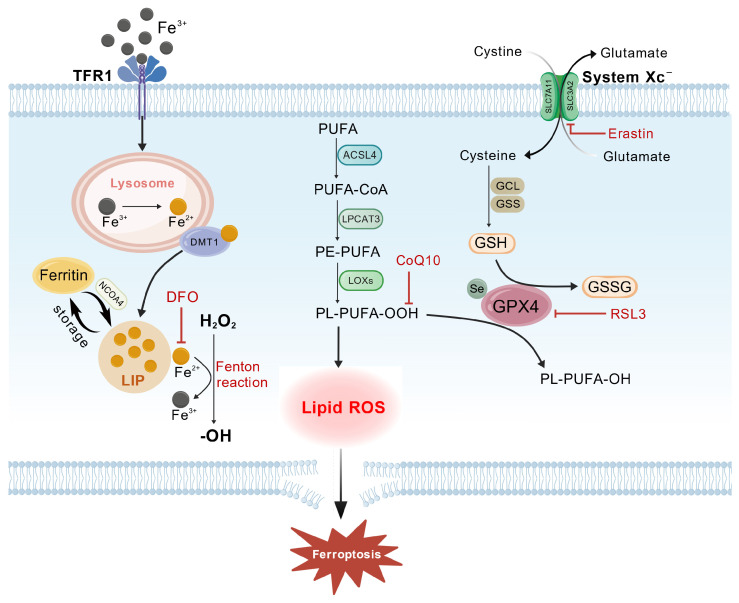
Core molecular mechanism of ferroptosis. Key pathways include: (1) Inhibition of the cystine/glutamate antiporter (System Xc-) reduces cystine uptake, depleting intracellular cysteine and GSH. (2) GSH depletion inactivates GPX4, the primary enzyme reducing toxic PL-PUFA-OOH to non-toxic phospholipid alcohols (PL-PUFA-OH). (3) Free labile iron (Fe^2+^) catalyzes the Fenton reaction, generating ROS that initiate/enhance peroxidation of polyunsaturated fatty acid (PUFA)-containing phospholipids. (4) Enzymes such as ACSL4 and LPCAT3 promote the incorporation of PUFAs into membrane phospholipids (PL-PUFA), making them susceptible to peroxidation. Unrestrained PL-PUFA-OOH accumulation leads to catastrophic membrane damage and cell death. Key ferroptosis inducers (e.g., erastin, RSL3) act at specific nodes as shown.

There is cellular uptake of iron through transferrin-bound iron (Fe^3+^), mediated through transferrin receptor 1 (*TFR1*). Fe^3+^ is also reduced to Fe^2+^ and delivered to the cytosol by divalent metal transporter 1 (*DMT1*) within lysosomes to constitute the labile iron pool (LIP). Although essential in iron homeostasis, excess absorption or storage defects increase the level of Fe^2+^ in the LIP [99,100]. The excess Fe^2+^ is usually stored as ferritin. Degradation of ferritin through ferritinophagy via Nuclear Receptor Coactivator 4 (*NCOA4*) releases Fe^2+^ to the LIP. This Fe^2+^ is a catalyst for the Fenton reaction. It catalyzes the conversion of H_2_O_2_ into highly reactive hydroxyl radicals (·OH), which directly oxidize membrane lipids, triggering lipid peroxidation (Figure 4) [101]. Fe^2+^ is involved in the generation of ·OH in the Fenton reaction of H_2_O_2_, which is a significant source of ROS. Also, leakage of electrons of mitochondrial electron transport chain complexes (I and III) produces ROS. These ROS coexist with Fe^2+^ to enhance oxidative injury, ultimately increasing lipid peroxidation and cell death [102,103]. Thus, iron dysregulation is a key inducer of ferroptosis, which itself represents a consequence of iron imbalance.

#### 4.2.2. Lipid Peroxidation

Lipid peroxidation is a key and an endpoint effector of ferroptosis. Polyunsaturated fatty acid (PUFA) oxidation produces products that undermine membrane integrity, increase permeability, disrupt ion homeostasis, and eventually impair organelles and trigger cell death. Lipid peroxidation refers to the oxidation of PUFAs of the membrane mediated by ROS, which results in the production of lipid hydroperoxides (LOOH). These LOOHs then react with additional lipids in the membrane, and propagative chain reactions ensue, continuing to propagate oxidation and causing massive cellular damage [104]. Long-chain PUFAs are converted to acyl-CoA esters (PUFA-CoA) by acyl-CoA synthetase long-chain family member 4 (*ACSL4*). Then, the lysophosphatidylcholine acyltransferase 3 (*LPCAT3*) esterifies these PUFA-CoAs into membrane phospholipids, especially phosphatidylethanolamine (PE). This alteration entraps PUFAs into cell membranes, making them the primary targets of peroxidation (Figure 4) [105,106].

Lipid peroxidation occurs through both enzymatic and non-enzymatic mechanisms. Specific enzymes catalyze enzymatic peroxidation. Lipoxygenases, like *ALOX15*, oxidize PUFA-PE directly. Using a non-heme iron-dependent proton-coupled electron transfer (PCET) mechanism, LOX selectively takes a hydrogen atom from the pentadiene center of PUFAs, making a lipid alkyl radical. After that, molecular oxygen is added to make LOOH [107]. Non-enzymatic peroxidation transpires independently of enzymes, predominantly initiated by radicals or reactive oxygen species (ROS). Fe^2+^ causes reactive radicals to form through the Fenton reaction. This starts autocatalytic lipid peroxidation chain reactions that make lipid peroxides (L-ROS). This pathway is the most important when GPX4 is blocked or GSH levels are low [98,108].

Therefore, lipid metabolism, through its dynamic regulation of the PUFA content in cell membrane phospholipids, serves as a key determinant of cellular susceptibility to ferroptosis. The pro-ferroptotic enzymes ACSL4 and LPCAT3 facilitate the incorporation of PUFAs into membrane phospholipids, thereby providing substrates for lipid peroxidation. In contrast, the anti-ferroptotic *SREBP1* pathway enhances cellular resistance by upregulating the synthesis of monounsaturated fatty acids (MUFAs), which competitively reduce the integration of PUFAs into the membrane. The balance between these two pathways constitutes a crucial mechanism by which lipid metabolism regulates the threshold for ferroptosis.

#### 4.2.3. Collapse of the Antioxidant Defense System

The induction of ferroptosis is fundamentally governed by the collapse of the GSH-GPX4 axis, which constitutes the primary and most critical regulatory hub in ferroptotic cell death. This axis operates through a hierarchical dependency: the cystine/glutamate antiporter (System Xc^−^, composed of *SLC7A11* and *SLC3A2*) is essential for importing extracellular cystine, which is subsequently reduced to cysteine—the rate-limiting precursor for the synthesis of glutathione (GSH). As a major cellular antioxidant and reducing agent, GSH specifically serves as the indispensable cofactor and electron donor for glutathione peroxidase 4 (GPX4). GPX4 is the only known enzyme capable of directly reducing toxic phospholipid hydroperoxides (PL-OOH) to their corresponding non-toxic phospholipid alcohols (PL-OH) within cell membranes. Therefore, the integrity of the System Xc^−^ → GSH → GPX4 axis is paramount for maintaining membrane lipid homeostasis and preventing lethal peroxidation. When this axis is compromised—whether through inhibition of System Xc^−^ (leading to GSH depletion) or direct inactivation of GPX4—the cell loses its ultimate defense against lipid peroxidation, resulting in the irreversible accumulation of peroxidized lipids, membrane damage, and the execution of ferroptosis [109,110,111]. Thus, the GSH-GPX4 axis is not merely one of several antioxidant pathways but represents the central and non-redundant regulatory node whose failure is the definitive trigger for ferroptosis.

### 4.3. Induction of Ferroptosis in Cancer Cells

Ferroptosis has emerged as a novel therapeutic target in cancer, with mechanistic research extending from basic biology to clinical translation. By targeting the system Xc^−^-GPX4 axis, iron metabolism, and lipid peroxidation pathways, various small-molecule drugs and natural compounds demonstrate significant anti-tumor efficacy.

#### 4.3.1. Small-Molecule Inducers

Small-molecule ferroptosis inducers are compounds that modulate intracellular ferroptosis-related pathways to induce this form of cell death. They regulate ferroptosis pathways through distinct mechanisms, including inhibiting GSH synthesis, directly suppressing GPX4 activity, modulating lipid metabolism, and altering iron ion concentration. These compounds have potential applications in cancer therapy, metabolic diseases, and neurodegenerative disorders, although further research into their toxicological profiles and clinical safety is warranted (Table 3).

#### 4.3.2. Genetic Manipulation

The application of genome-editing technologies to induce ferroptosis levels is primarily based on targeting specific genes or signaling pathways to study and regulate ferroptotic pathways, thereby providing new therapeutic approaches for cancer. *ACSL4* is a central favorable regulator of ferroptosis that enhances lipid peroxidation vulnerability by promoting the incorporation of polyunsaturated fatty acids into membrane phospholipids. The expression of *ACSL4* via gene editing has been reported to significantly sensitize cancer cells to radiotherapy and ferroptosis-inducing agents [121,122]. The *SLC7A11*/*GPX4* signaling is one of the core inhibitory signals in the mechanism of ferroptosis. The impaired absorption of cystine by the impairment of *SLC7A11* leads to a reduction in glutathione levels, which is accompanied by the direct inhibition of GPX4, affecting the ability of cells to eliminate lipid peroxides. CRISPR-mediated knockout of the *SLC7A11* or *GPX4* genes has demonstrated pro-ferroptotic effects in various models, including liver cancer and lung cancer (Table 4) [123,124].

## 5. Interactive Mechanisms Between MDR and Ferroptosis in Colon Cancer

### 5.1. Evidence of Crosstalk Between MDR and Ferroptosis Pathways

Multilayered evidence demonstrates crosstalk between MDR and ferroptosis pathways, involving metabolic, transcriptional, oxidative stress, and signaling interactions.

#### 5.1.1. Interaction of Drug Efflux Pumps with Ferroptosis Inducers

Sorafenib, a kinase inhibitor, induces ferroptosis by inhibiting System Xc^−^. However, tumor cells with high P-gp expression exhibit significantly reduced sensitivity to sorafenib, resulting in efflux pump-mediated resistance. For instance, UO-31 renal carcinoma cells regain sensitivity to ferroptosis inducers in the presence of the P-gp inhibitor valsodar, directly confirming P-gp-mediated efflux of ferroptosis inducers [132]. Sorafenib metabolism involves not only P-gp but also transporters like OATP, MRP-2, and BCRP. This multi-pathway efflux may synergistically reduce intracellular drug concentrations, establishing cross-resistance to ferroptosis [133].

#### 5.1.2. Overlap in Antioxidant Defense Systems

**Figure 5:**
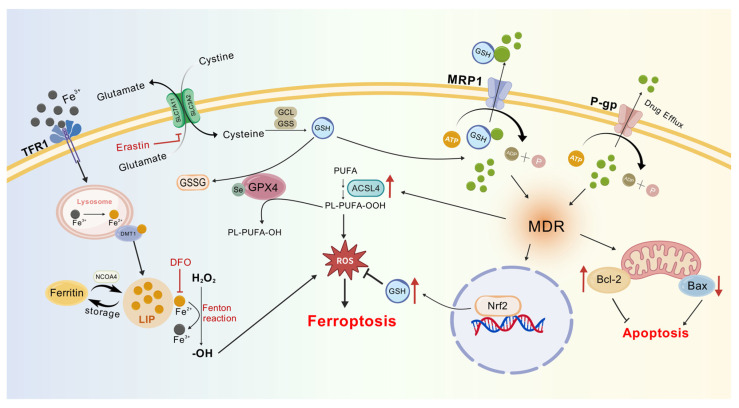
Interplay between ferroptosis and MDR pathways in cancer.

Significant overlap exists between core ferroptosis regulators (GPX4, System Xc^−^), and the MDR antioxidant system is what enables dual resistance at the molecular level. GPX4 lowers lipid peroxides (PL-OOH) in a way that depends on GSH, and MDR cells avoid ferroptosis by increasing GPX4 or making more GSH. For instance, when Nrf2 is activated, it increases the activity of GCL (glutamate-cysteine ligase) and GSS, raising GSH levels [134,135]. System Xc^−^ inhibitors deplete GSH by blocking cystine uptake, but System Xc^−^ overexpression in MDR cells sustains GSH levels, conferring resistance to both chemotherapeutics and ferroptosis (Figure 5). Clinical data demonstrate that sorafenib inhibits System Xc^−^ activity. Yet resistant tumors evade this by upregulating *SLC7A11* (a System Xc^−^ subunit) [136].

#### 5.1.3. Dual Regulation by the Nrf2 Signaling Pathway

*Nrf2* (Nuclear factor erythroid 2-related factor 2), the master regulator of the antioxidant response, plays a pivotal role in both MDR and ferroptosis suppression. The *Nrf2* pathway exerts a dual influence on antioxidant defense and chemoresistance. On one hand, *Nrf2* enhances cellular resistance to oxidative stress by activating downstream genes (e.g., *GPX4*, *SLC7A11*, *GCL*), protecting cells from oxidative damage [135,137]. On the other hand, while hyperactivation of *Nrf2* inhibits ferroptosis, it also promotes chemoresistance. Studies show that *Nrf2* protects cancer cells from chemotherapy toxicity by inhibiting drug-induced apoptosis and oxidative stress responses. For instance, in the context of doxorubicin (DOX) resistance, *Nrf2* enhances cancer cell survival by upregulating antioxidant and detoxifying enzymes, thereby decreasing drug toxicity [135]. Furthermore, *Nrf2* is also involved in changing the immune system in the tumor microenvironment. This could make chemoresistance even more pronounced by reducing inflammation and increasing the number of cancer stem cells [138,139].

#### 5.1.4. Dynamic Regulation of Iron Metabolism

Iron homeostasis imbalance is a core trigger of ferroptosis, and MDR cells establish dual barriers by modulating iron uptake, storage, and efflux. MDR cells downregulate *TFRC* to reduce iron import while upregulating ferritin to sequester labile iron, suppressing lipid peroxidation. For example, high ferritin expression correlates with chemoresistance in ovarian tumor-initiating cells (OTICs). Upregulation of ferroportin may further decrease intracellular iron levels, forming a dual resistance barrier [140].

### 5.2. Impact of MDR on Ferroptosis Susceptibility

MDR cells reduce ROS accumulation by activating transcription factors such as *Nrf2* and *HIF-1α*, thereby upregulating antioxidant molecules, including GSH and SOD. For instance, prolonged oxidative stress exposure in MCF-7 cells significantly enhanced ferroptosis resistance via *Nrf2* activation [141]. Additionally, MDR cells may attenuate ROS generation by reducing mitochondrial biogenesis (e.g., decreased mitochondrial membrane potential in drug-resistant osteosarcoma cells) [142]. MDR cells can also suppress lipid peroxidation by upregulating ferritin and hepcidin to increase iron storage, reducing the Fenton reaction activity of labile iron. Expression of mitochondrial iron exporters like *ABCB8* may be suppressed, diminishing intramitochondrial iron retention and weakening iron-dependent oxidative damage [143,144]. MDR is further linked to alterations in lipid metabolism. Studies indicate that accumulation of specific lipids (e.g., glucosylceramide in MDR breast cancer cells) may alter membrane fluidity or inhibit lipid peroxidation chain reactions, thereby enhancing resistance [145]. MDR cells may reduce the PUFA ratio or increase monounsaturated fatty acids (MUFAs), lowering lipid peroxidation propensity and conferring ferroptosis resistance [146].

### 5.3. Role of Ferroptosis in Overcoming MDR

Ferroptosis represents an innovative strategy to overcome MDR. Its fundamental advantage lies in the essential distinction of its mechanism of action from that of traditional chemotherapeutic agents and apoptosis inducers, enabling it to circumvent the primary defense systems upon which MDR cells rely and to exploit the inherent metabolic vulnerabilities of tumor cells for selective targeting.

Firstly, ferroptosis bypasses apoptosis resistance pathways, targeting cell populations deemed “un-killable”. Conventional therapies primarily function by activating apoptotic pathways, while MDR cells often acquire robust resistance through mechanisms such as upregulating Bcl-2 family proteins or mutating *p53*. Ferroptosis, in contrast, is a programmed cell death modality independent of caspase activation. It directly attacks and disrupts the lipid bilayer structure of cell membranes through the accumulation of iron-dependent lipid peroxidation, leading to loss of membrane integrity. Consequently, ferroptosis inducers remain effective in inducing cell death even in tumor cells with defective apoptotic pathways. For example, *GPX4* inhibitors can selectively kill drug-resistant ovarian and lung carcinoma cells, even in the presence of *Bcl-2* overexpression or *p53* mutation [147,148].

Secondly, the mechanism of ferroptosis induction evades drug efflux mediated by ABC transporters. The action of many ferroptosis inducers does not rely on achieving high intracellular concentrations; instead, they initiate the cell death program by irreversibly inhibiting key targets (e.g., *GPX4*) or interfering with metabolic processes (e.g., depleting GSH). Therefore, these inducers are typically not efficient substrates for ABC transporters. For instance, erastin promotes lipid peroxidation by inhibiting the cystine/glutamate antiporter (System Xc^−^), a process not mediated by the P-gp efflux system. Clinical studies have demonstrated that sorafenib overcomes therapeutic resistance in hepatocellular carcinoma through a similar mechanism [149,150].

Finally, ferroptosis strategies exploit the metabolic weakness of tumor cells—“iron addiction”—for selective killing. Rapidly proliferating tumor cells have a significantly increased demand for iron to support their metabolism, manifested as an expanded intracellular labile iron pool. This “iron-addicted” state renders them more susceptible to ferroptosis. Ferroptosis induction strategies precisely exploit this metabolic imbalance: by exacerbating intracellular iron overload to catalyze the Fenton reaction, they drive lethal lipid peroxidation. In contrast, normal cells maintain relatively stable iron homeostasis and are consequently less sensitive. Research indicates that combining iron chelators with traditional chemotherapy can further increase the ferroptosis susceptibility of MDR cells [151,152].

### 5.4. Molecular Targets Linking MDR and Ferroptosis

#### 5.4.1. ROS Regulation

One of the key characteristics of ferroptosis is the accumulation of reactive oxygen species, which is caused by iron-dependent lipid peroxidation. Multidrug-resistant (MDR) cells often overcome the cytotoxic effect of chemotherapy by increasing antioxidant defenses.

A selenium-dependent glutathione peroxidase 4 (GPX4) works to stop the lipid peroxidation chain reaction by reducing the lipid hydroperoxides (LOOH) to the corresponding non-toxic lipid alcohols (LOH). It depends directly on the presence of glutathione (GSH) as a cofactor, and GSH production depends on cystine uptake via the xCT transporter. The impairment of GPX4 activity triggers mitochondrial metabolic dysfunction, reduced ATP production, and neuronal death, highlighting its essential role in maintaining basic cellular metabolism [153,154]. The overexpression of *GPX4* in MDR cells confers resistance to chemotherapeutic agents by reducing ferroptosis; elevated enzyme activity maintains intracellular redox balance, reduces ROS-induced toxicity, and enhances the survival of resistant cells. Pharmacological inhibition of GPX4 returns drug resistance to its original state by either directly inhibiting its catalytic activity or removing GSH, thereby inducing ferroptosis. In recent case studies, *GPX4* knock-out or knockdown selectively removes resistant cell populations in models of ovarian and lung cancer and inhibits tumor recurrence [155].

The master transcription factor regulator of the antioxidant response is nuclear factor erythroid 2-related factor 2 (*Nrf2*). When activated, it induces the expression of genes such as xCT and *GPX4*, thereby enhancing GSH production and lipid peroxide clearance. Its activity is regulated by KEAP1-mediated ubiquitination and degradation, and it can be activated by oxidative stress or oncogenic signaling [130,156]. *Nrf2* activation boosts the antioxidant capacity of MDR cells by upregulating xCT and *GPX4*, reducing the cytotoxicity of chemotherapeutic drugs. For example, in non-small-cell lung cancer, activation of the Nrf2-xCT axis directly correlates with cisplatin resistance [157,158]. Nrf2 suppresses lipid peroxide accumulation by maintaining GSH levels and GPX4 activity. *Nrf2* deficiency or inhibition weakens antioxidant defenses, sensitizing cells to ferroptosis inducers [159]. Inhibiting *Nrf2* can synergize with chemotherapeutic drugs to induce ferroptosis. For instance, atorvastatin overcomes hepatocellular carcinoma cell resistance by downregulating *Nrf2* and its target genes, triggering mitochondrial-dependent ferroptosis [160].

xCT is the catalytic subunit (*SLC7A11*) of system Xc^−^, forming a heterodimer with *SLC3A2* to mediate the 1:1 exchange of extracellular cystine for intracellular glutamate. Cystine is reduced to cysteine for GSH synthesis, supporting GPX4 function. xCT may promote cell death under glucose starvation, revealing its dual role in metabolic stress [161,162]. xCT overexpression significantly elevates GSH levels, enhancing the resistance of MDR cells to chemotherapeutic drugs. Clinical data demonstrate that elevated xCT expression is associated with poor prognosis in patients with glioma and colorectal cancer [163]. Blocking xCT stops cystine uptake, lowers GSH levels, inactivates GPX4, and causes lipid peroxide to build up, which subsequently leads to ferroptosis. For instance, erastin permanently inhibits xCT transport by binding to its TM6b domain. This makes cisplatin more cytotoxic against ovarian cancer cells that are resistant to it [164,165].

#### 5.4.2. Iron Homeostasis

Iron metabolism dysregulation is necessary for ferroptosis, and multidrug-resistant (MDR) cells can influence drug sensitivity by altering iron uptake, storage, and release.

Transferrin receptor 1 (*TFRC*) is a vital receptor that assists cellular iron uptake. It initiates endocytosis by binding to transferrin-bound iron, releasing labile iron into the cytosol. Iron overload accelerates the Fenton reaction, generating ROS that induce lipid peroxidation and ferroptosis. MDR cells frequently downregulate *TFRC* to diminish iron import and mitigate the risk of ferroptosis. For instance, in colon cancer cells, stimulating *TFRC* through β-catenin signaling enhances iron accumulation and their capability to repair DNA damage, thereby rendering the cells more resistant. In colon cancer models, the combination of *TFRC* inhibitors and DNA-damaging agents enhances replicative stress and reduces tumor growth [166,167]. Targeting *TFRC* elevates intracellular iron accumulation, thereby restoring chemosensitivity. For example, miR-497-5p significantly inhibits cervical cancer cell proliferation and migration by suppressing *TFRC* expression; anti-TFR1 antibodies or transferrin-conjugated drugs increase tumor-specific cytotoxicity [152,168].

Ferritin, which consists of *FTH1* and *FTL* subunits, helps keep iron from being toxic by storing it. Ferritinophagy, regulated by *NCOA4*, degrades ferritin and releases iron into the cytosol, leading to ferroptosis [169]. Ferritin overexpression safeguards cells by chelating iron, thereby inhibiting ferroptosis. For instance, in resistant colorectal cancer cells, lipocalin 2 (LCN2) lowers iron levels by increasing ferritin and *GPX4* levels, which makes the cells resistant to 5-FU [170]. Activating *NCOA4*-dependent ferritinophagy liberates iron and triggers ferroptosis. Inhibiting autophagy or knocking out *NCOA4* decreases iron release and stops ferroptosis. Iron chelators also stop ferroptosis by stopping iron release, but they may make cells more sensitive to chemotherapy [171,172].

Hepcidin is a hormone made by the liver that controls iron levels. It binds to ferroportin (FPN), which exports iron from the body. This causes FPN to be tagged for destruction, which lowers iron export. Hepcidin tightly controls FPN’s activity, making it the only iron exporter in mammals. When hepcidin levels increase, FPN levels decrease. This keeps iron inside cells and accelerates lipid peroxidation. Nonetheless, MDR cells may equilibrate iron toxicity and ensure survival by stimulating antioxidant pathways. For example, long-term inflammation increases Hepcidin through the *IL-6*/*JAK*/*STAT3* pathway, which makes breast cancer and glioma more resistant [173,174]. Blocking Hepcidin or activating FPN raises iron levels, while blocking FPN lowers them. Hepcidin antagonists or FPN agonists exhibit promise in clinical trials [175].

#### 5.4.3. Lipid Metabolism

*ACSL4* catalyzes the esterification of PUFAs such as arachidonic acid (AA) and adrenic acid (AdA), thereby incorporating them into membrane phospholipids. This change makes membrane lipids much more likely to undergo peroxidation, which is a significant cause of ferroptosis. The ferroptosis threshold is directly affected by *ACSL4* activity. High expression of *ACSL4* accelerates lipid peroxidation, whereas knocking it down makes cells more resistant to ferroptosis [129]. Downregulation of *ACSL4* in resistant cells reduces PUFA-phosphatidylethanolamine (PUFA-PE) generation, thereby lowering lipid peroxidation levels and enhancing ferroptosis resistance [176]. *ACSL4* also upregulates ABC transporter expression by activating the *mTORC1/2* signaling pathway, which promotes the efflux of chemotherapeutic drugs [177].

LOXs (Lipoxygenases) directly catalyze PUFA oxidation to generate LOOH, acting as executioners of ferroptosis. Specifically, 15-LOX, upon binding phosphatidylethanolamine-binding protein 1 (PEBP1), oxidizes PUFAs within membrane phospholipids, generating pro-ferroptotic OOH-PE signals. However, the role of LOXs is cell-type-dependent, and auto-oxidation (non-enzymatic reactions) may predominate in some contexts [178]. LOX inhibitors block ferroptosis by reducing LOOH accumulation and enhancing tumor cell resistance to chemotherapy [179]. Some resistant cells upregulate LOX activity to promote lipid peroxidation but achieve compensatory protection by enhancing the GPX4 or FSP1/CoQ10 axis [180].

*SREBP1* (Sterol regulatory element-binding protein 1) is a master transcription factor for lipid synthesis, regulating genes like fatty acid synthase (*FASN*) and acetyl-CoA carboxylase (*ACC*). Its activation increases the proportion of saturated fatty acids (SFAs) and MUFAs, reducing membrane lipid unsaturation and thereby inhibiting lipid peroxidation [181,182]. *SREBP1* activation promotes lipid raft formation, enhances membrane fluidity, facilitates ABC transporter membrane localization and drug efflux; MUFA accumulation reduces ferroptosis sensitivity by competitively inhibiting PUFA incorporation into membrane phospholipids [183].

An in-depth investigation of these molecular targets provides new strategies to reverse MDR and enhance sensitivity to ferroptosis. MDR and ferroptosis create a complicated regulatory network with important molecular targets such as ROS regulation, iron homeostasis, and lipid metabolism. This gives novel concepts for overcoming resistance. GPX4 inhibits ferroptosis by lowering lipid peroxides. Its elevated levels are linked to MDR, and blocking *GPX4* or targeting the *NRF2-xCT* pathway can disrupt antioxidant defenses and restore chemosensitivity. Iron homeostasis is affected by *TFRC*-mediated iron uptake, ferritin-mediated iron storage, and the Hepcidin-FPN axis-mediated iron export. Targeting these molecules can cause iron overload, which leads to lipid peroxidation, ferroptosis, and overcoming resistance. *ACSL4* and LOXs are responsible for PUFA esterification and oxidation, respectively, in lipid metabolism. Changes in their activity are closely linked to MDR, and lipid synthesis reprogramming mediated by *SREBP1* further reinforces the resistant phenotype. Changing these targets can make cells more sensitive to lipid peroxidation and cooperate with chemotherapy to kill resistant cells. Based on these mechanisms, strategies that use GPX4 inhibitors, iron metabolism interventions, and lipid metabolism modulators together could break the MDR bottleneck by causing ferroptosis. This could lead to new ways to treat cancer.

## 6. New Therapeutic Perspectives

### 6.1. Combination of Ferroptosis Inducers with Chemotherapy

Chemotherapeutic agents eradicate tumor cells by causing DNA damage or disrupting metabolic processes; however, multidrug-resistant (MDR) cells frequently evade apoptosis by bolstering antioxidant defenses or diminishing lipid peroxidation. Ferroptosis inducers directly disrupt the antioxidant barrier of MDR cells by inhibiting the xCT/GPX4 axis or increasing iron accumulation, thereby amplifying chemotherapy cytotoxicity. In A549 lung cancer and HCT116 colon cancer cells, the combination of cisplatin and Erastin decreased cell viability to 15%, which is much lower than either drug alone. Erastin inhibits GSH synthesis, preventing the effective clearance of cisplatin-induced ROS, leading to exacerbated DNA damage [184]. Furthermore, utilizing iron delivery systems (e.g., TBP-Ps vesicles, Fe^3+^-citrate complexes) to increase intracellular labile iron levels effectively promotes the conversion of chemotherapy-generated ROS into lethal lipid peroxides, breaching the antioxidant threshold of MDR cells. In resistant colon and breast cancer cells and models, iron delivery systems combined with doxorubicin significantly enhanced drug uptake and tumor suppression rates, achieving far superior tumor regression compared to monotherapy, with good safety profiles [185,186,187].

### 6.2. Combination of Ferroptosis Inducers with Targeted Therapy

Targeted drugs may activate pro-survival signals, indirectly enhancing MDR; ferroptosis inducers can achieve synergistic killing by blocking these pathways or targeting downstream effectors. GPX4 inhibitors directly block the reduction of lipid peroxides, counteracting *Nrf2*-mediated *GPX4* upregulation [188]. Osimertinib inhibits EGFR signaling, reducing tumor proliferation, but may activate the *Nrf2/GPX4* pathway; RSL3 directly inhibits GPX4, causing irreversible accumulation of lipid peroxides, synergistically overcoming resistance [189]. PARP inhibitors induce DNA damage, relying on synthetic lethality in BRCA-deficient cells; FIN56 induces ferroptosis via dual mechanisms (GPX4 degradation and CoQ10 depletion), achieving a “dual hit” in repair-deficient cells [190]. FIN56 activates squalene synthase (SQS), depleting CoQ10 and enhancing lipid ROS accumulation, synergizing with the DNA-damaging effects of PARP inhibitors [191].

### 6.3. Synergy of Ferroptosis Inducers with Immunotherapy

Ferroptosis releases tumor antigens and damage-associated molecular patterns (DAMPs), activating dendritic cells (DCs) and cytotoxic T cells, thereby enhancing the efficacy of immune checkpoint inhibitors. Immunotherapy, on the other hand, can reverse immunosuppression in the tumor microenvironment (TME), which leads to ferroptosis and a positive feedback loop [192,193]. Sorafenib, an inhibitor of System Xc^−^, prevents the uptake of cystine, which decreases the production of GSH and inactivates GPX4. This causes lipid ROS to build up and ferroptosis to happen. At the same time, sorafenib alters the interaction between BECN1 and *SLC7A11* via the *SHP-1*/*STAT3* signaling pathway, further slowing cystine metabolism [150]. Antigens and DAMPs released during ferroptosis activate dendritic cells (DCs), which makes tumors more immunogenic [194]. Anti-PD-1 antibodies relieve T-cell inhibition, promoting their infiltration and cytotoxic function, while also suppressing *SLC7A11* via *IFN-γ* feedback [195]. This combination significantly inhibited tumor growth in hepatocellular carcinoma (HCC) models, and patient serum oxidative markers correlated with improved prognosis [149].

As an iron chelator, deferoxamine (DFO) reduces the labile iron pool (LIP) by binding free iron ions while simultaneously upregulating *TFR1* on tumor cells, thereby increasing iron-uptake-dependent lipid peroxidation. DFO also inhibits DNA synthesis and epithelial–mesenchymal transition (EMT), sensitizing tumors to CAR-T therapy. The iron homeostasis imbalance induced by DFO makes tumor cells more dependent on GPX4, and the targeted killing by CAR-T cells further depletes GPX4 reserves, exacerbating ferroptosis. In hematological and solid tumor models, the combination of DFO and CAR-T overcame tumor resistance and significantly prolonged survival [196,197].

### 6.4. Developing Novel Drugs Targeting Both MDR and Ferroptosis

Developing bifunctional drugs that simultaneously target both pathways can overcome the limitations of conventional therapies through synergistic action. Such agents can restore chemosensitivity by inhibiting ABC transporters or lysosomal drug sequestration while concurrently triggering ferroptosis via GSH depletion, GPX4 inhibition, or activation of lipid peroxidation, thereby achieving a “dual-killing” effect. Pharmacophore fusion and linker design involve connecting a P-gp inhibitor and a ferroptosis inducer via a cleavable linker. Sorafenib, a multi-kinase inhibitor with trifluoromethyl, chlorophenyl, and amide groups, has a wide range of effects on tumors. A flexible spacer can connect RSL3, a covalent GPX4 inhibitor with a chloroacetamide group, to the pyridine ring of sorafenib. This keeps the important pharmacophores of both molecules [115].

Natural products are perfect for these kinds of drugs due to their inherent multi-target properties. For example, berberine stops P-gp from working, which reverses MDR, and at the same time, it stops the *Nrf2* pathway, which lowers GSH synthesis, which leads to ferroptosis [198,199]. Artemisinin promotes lipid peroxidation through iron-dependent ROS bursts while downregulating *ABCB1* expression to enhance chemotherapeutic drug accumulation [200]. Additionally, flavonoid-based bifunctional molecules can stop P-gp and promote lipid peroxidation at the same time [201]. These natural molecules not only offer robust chemical frameworks but can also be refined for improved selectivity and efficacy while minimizing off-target toxicity via structural modification or nanodelivery technologies.

### 6.5. Personalized Medicine Approaches

#### 6.5.1. Precision Targeting Based on Molecular Subtyping

Identifying mutations that confer resistance using whole-exome sequencing (WES) or RNA sequencing makes it easier to switch to targeted agents. WES primarily identifies mutations in coding regions, enabling the detection of gene variants linked to drug resistance. For instance, in patients with advanced non-small-cell lung cancer (NSCLC), whole-exome sequencing (WES) analysis identified resistance mechanisms, including tumor clonal selection, molecular alterations in Wnt pathway genes, and increased copy-number variations of cancer-related genes, which guided the application of third-generation targeted drugs such as osimertinib [202].

ctDNA liquid biopsy shows great promise for clinical use in tracking resistance mutations over time. Identifying the T790M resistance mutation through liquid biopsy in NSCLC patients predicts the mechanisms of resistance after EGFR-TKI treatment failure and informs subsequent therapeutic approaches [203]. Liquid biopsy has the benefits of being minimally invasive and being able to be performed in real time, enabling continuous monitoring of tumor molecular landscape evolution, capturing early signs of resistance, and making changes to personalized treatment plans.

#### 6.5.2. Metabolic and Microenvironment Intervention

Combination therapy with 2-DG (a glycolysis inhibitor) and CB-839 (a glutaminase inhibitor) exhibits strong synergistic antitumor effects in breast cancer cells, inducing apoptosis rates of up to 70%, particularly in resistant subtypes such as MCF7-TR and T47D-TR [204]. Choosing personalized inhibitors based on metabolomics can make them even more effective. Pirfenidone, an anti-fibrotic drug, blocks TGF-β signaling. This lowers the tumor’s elastic modulus and interstitial fluid pressure, which makes it easier for chemotherapy drugs to get into the tumor [205]. HIF-1α controls metabolic pathways like glycolysis and oxidative phosphorylation. HIF-1α inhibitors can boost ROS production by targeting these pathways, making chemotherapy work even better, as shown in colon cancer models [206].

#### 6.5.3. Nanotechnology and Smart Delivery

Liposomes or polymeric nanoparticles circumvent ABC efflux pumps to target chemotherapeutics directly to resistant cells. Liposomal doxorubicin, altered with polyethylene glycol (PEG), demonstrates extended circulation duration in the bloodstream, diminished clearance by the liver and spleen, and subsequently heightened accumulation at tumor locations. This greatly increases the effectiveness of drugs while lowering their cardiotoxicity [207]. pH-responsive nanoparticles use the acidic nature of the TME to release drugs directly at the tumor site, which reduces the risk of harming healthy tissues [208].

#### 6.5.4. Artificial Intelligence (AI) and Dynamic Monitoring

AI combines genomic, clinical, and imaging data to estimate the likelihood that a patient will be resistant. The OncoKB database method uses machine learning models and deep integration of multi-omics data to predict resistance risk. This method uses advanced computer models on multi-layered bioinformatics data to make accurate predictions about how cancer drugs will work. The DELFOS model-based drug-sensitivity prediction achieved 89% accuracy, which is much better than older methods [209,210].

CTC (Circulating Tumor Cell) and ctDNA (Circulating Tumor DNA) monitoring systems for dynamically tracking the evolution of resistant clones signify a novel liquid biopsy-based methodology. This system enables real-time evaluation of resistance mechanisms and evolving alterations in tumor clones during real-time evaluation of resistance mechanisms and the evolving alterations in tumor clones throughout cancer therapy. By analyzing cell-free DNA or cells in blood samples, it offers detailed information on tumor heterogeneity, the emergence of resistant clones, and the response to therapy [211].

#### 6.5.5. Clinical Trials and Future Directions

Ferroptosis inducers have demonstrated efficacy in multiple resistant tumor models. However, their low selectivity and poor pharmacokinetic properties make them dangerous to normal tissue if administered directly. Ferroptosis offers an innovative approach to circumvent conventional resistance mechanisms in multidrug-resistant (MDR) diseases; however, its clinical application faces obstacles such as mechanistic complexity, limitations in drug delivery, and safety concerns. To move forward with future advancements, it is necessary to work collaboratively across many fields to improve targeted delivery systems, combination therapies, and precision monitoring tools. Additionally, basic research needs to be intensified to better understand the dynamic regulatory network of ferroptosis. With the progress of many preclinical studies, ferroptosis is likely to begin early-stage clinical trials in the next 5 to 10 years. It will be a key way to solve the MDR problem [212,213].

## 7. Challenges and Limitations

### 7.1. Selectivity and Toxicity of Ferroptosis Inducers

Ferroptosis inducers frequently induce toxicity in normal tissues owing to inadequate targeting. Current prevalent inducers primarily function through system Xc^−^ or GPX4. However, because these targets are found in so many normal cells, drugs may not be able to target them specifically, which could cause systemic toxicities like hepatotoxicity and nephrotoxicity [214,215].

To solve these problems, prodrugs that respond to the tumor microenvironment can be developed. These drugs use the tumor’s low pH, high ROS, or specific enzymatic environment to make them more selective. Targeted delivery systems, like nanocarriers or antibody-drug conjugates (ADCs), can also be used to make drugs more concentrated in tumor tissues [216].

### 7.2. Tumor Heterogeneity and Resistance to Ferroptosis

Tumor heterogeneity refers to differences in genotype, phenotype, and metabolic characteristics among distinct cell subpopulations within a tumor, leading to inconsistent responses to ferroptosis. Different subpopulations may harbor mutations affecting key ferroptosis pathways, such as *p53* mutations or variations in GPX4 or xCT expression levels [217,218]. Some subpopulations remodel lipid metabolism to reduce lipid peroxidation or rely on the *GCH1*-BH4 pathway to selectively protect polyunsaturated fatty acid phospholipids (PUFA-PLs), enhancing ferroptosis resistance [219]. CSCs are a major source of tumor heterogeneity. CSCs exhibit high expression of ABC transporters, which efflux ferroptosis inducers and reduce intracellular drug concentrations; they activate repair pathways (e.g., *p53*, *ATM*) to gain time for repair through cell cycle arrest; and the hypoxic microenvironment further suppresses ferroptosis [220].

### 7.3. Translating Preclinical Research to Clinical Applications

Traditional in vitro cell lines and mouse xenograft models fail to recapitulate the complex tumor microenvironment and immune interactions of human tumors, particularly as immunodeficient mice cannot simulate the role of the immune system [221]. There is a lack of humanized models that accurately reproduce tumor heterogeneity and therapeutic resistance. Immortalized cell lines lose primary tumor heterogeneity due to long-term in vitro culture, while patient-derived xenograft (PDX) models partially retain heterogeneity but are costly, time-consuming, and have low success rates. Humanized immune mice or orthotopic transplantation models can better simulate the authentic immune microenvironment [222]. Three-dimensional organoids derived from fresh patient tumor tissue, preserving genetic heterogeneity and microenvironmental features, offer a more reliable preclinical model [223].

Species differences often limit the stability, bioavailability, and tissue penetration of ferroptosis inducers. For instance, variations in drug-metabolizing enzyme and transporter expression between mice and humans may preclude extrapolation of preclinical data to patients. Engineered nanocarriers or liposomes can improve drug targeting and penetration while reducing off-target effects [224]. At present, there is an absence of dependable clinical biomarkers for the real-time monitoring of ferroptosis induction. The conventional biomarker discovery pipeline encounters obstacles such as technical translation challenges and validation issues [225].

## 8. Conclusions

The fundamental mechanisms of multidrug resistance (MDR) in colon cancer involve multiple levels, including the overexpression of drug transporters, enhanced metabolism and detoxification, defective DNA repair, and epigenetic regulation. Ferroptosis is a type of controlled cell death that depends on iron and is caused by the buildup of lipid peroxides. Important steps in this process include the esterification of PUFAs by *ACSL4*/*LPCAT3*, the depletion of GSH due to the inhibition of System Xc^−^, and the inactivation of GPX4. Notably, crosstalk exists between MDR and ferroptosis: MDR-associated pathways can suppress ferroptosis, ABC transporters can efflux ferroptosis inducers, and dysregulated iron metabolism further diminishes ferroptosis susceptibility. To overcome MDR, novel strategies are being developed, such as the combination of ferroptosis inducers with chemotherapy or targeted agents to circumvent apoptosis resistance, the use of natural products for dual-targeting interventions, and the application of precision medicine informed by molecular subtyping or artificial intelligence models.

Understanding how MDR and ferroptosis work together is important for overcoming the limits of traditional treatments. MDR and ferroptosis establish a bidirectional regulatory network by sharing nodes in iron homeostasis, antioxidant defense, and lipid metabolism. A key advantage of ferroptosis is that it does not rely on traditional apoptotic pathways. This means it can kill cells that are resistant to apoptosis, such as those with Bcl-2 overexpression or *p53* mutations. Mechanistically, inhibiting the Nrf2-xCT-GPX4 axis simultaneously weakens the core antioxidant defense of MDR and triggers ferroptosis; altering iron homeostasis may increase lipid peroxidation, which synergizes with the ROS toxicity of chemotherapy drugs. For clinical translation, combination therapies harness ferroptosis to liberate tumor antigens and damage-associated molecular patterns (DAMPs), thereby eliciting anti-tumor immune responses. Concurrently, nanodelivery systems facilitate the precise targeting of iron ions or pharmaceuticals to tumor locations, markedly diminishing systemic toxicity and expanding the therapeutic window.

To accelerate the clinical translation of ferroptosis as an anti-MDR strategy, future work must establish a closed-loop research pathway: Mechanistically, in-depth elucidation of how tumor heterogeneity and microenvironmental factors regulate the MDR-ferroptosis dynamic equilibrium is essential, particularly focusing on HIF-1α’s role in coordinating iron metabolism and drug resistance. Technological advancements are needed to develop microenvironment-responsive delivery systems that enhance the selectivity of ferroptosis inducers and to identify specific biomarkers, such as lipid peroxide derivatives, for real-time assessment of efficacy. To improve clinical translation, it is essential to look into how ferroptosis inducers and metabolic interventions can work together, use ctDNA/CTC multi-omics data to help personalize therapy, and use 3D organoids or immuno-humanized mouse models to get around the problems that come up in preclinical research. At the same time, by developing specific biomarkers and bifunctional drugs targeting *GPX4*/*ACSL4*, the bottlenecks of safety and efficacy can ultimately be addressed, enabling precise clinical translation.

## Figures and Tables

**Table 1:** Composition and functions of the ABC transporter family.

Subfamily	Representative Members	Primary Functions/Substrates	Associated Diseases or Phenotypes	References
ABCA	*ABCA1*	Reverse cholesterol transport, HDL formation	Tangier disease, Atherosclerosis	[15]
ABCB	*ABCB1* (MDR1/P-gp)	Efflux of multiple drugs (chemotherapeutics, toxins)	Cancer multidrug resistance	[16]
	*ABCB2/B3* (TAP1/TAP2)	Antigenic peptide transport to MHC-I molecules	Immunodeficiency	[17]
ABCC	*ABCC1* (MRP1)	Transport of glutathione-conjugated drugs	Tumor drug resistance, Dubin-Johnson syndrome	[18]
ABCD	*ABCD1*	Transport of very long-chain fatty acids (VLCFAs) to peroxisomes	X-linked adrenoleukodystrophy (X-ALD)	[19,20]
ABCE	*ABCE1*	Ribosome metabolism, Non-transport function	Regulation of viral infection	[15,21]
ABCF	*ABCF1*	Translation regulation, Non-transport function	Immune regulation	[15,21]
ABCG	*ABCG2* (BCRP)	Drug/urate transport	Gout, Chemotherapy resistance	[22]
	*ABCG5/G8*	Efflux of plant sterols (phytosterols)	Sitosterolemia, Gallstone disease	[15]

**Table 2:** Differences between ferroptosis and apoptosis.

Characteristics	Ferroptosis	Apoptosis	References
Core Mechanism	Iron-dependent accumulation of lipid peroxides	Activation of the Caspase protease family	[97]
Cellular Morphology	Mitochondrial shrinkage, reduction or disappearance of cristae, absence of apoptotic bodies	Cell shrinkage, chromatin condensation, formation of apoptotic bodies	[97]
Key Molecules	GPX4, System Xc^−^ (SLC7A11), ACSL4, free iron (Fe^2+^)	Caspases, Bcl-2 family proteins, p53	[97]
Dependency	Iron-dependent	ATP-dependent	[97]

**Table 3:** Common small-molecule ferroptosis inducers.

Category	Representative Agents	Target	Mechanism	Applicable Cancers	References
System Xc^−^ Inhibitors	Erastin, Sorafenib	*SLC7A11/SLC3A2*	Block cystine uptake → GSH depletion → GPX4 inactivation → Lipid peroxide accumulation	Liver cancer, Renal cancer, Glioblastoma	[112,113,114]
GPX4 Direct Inhibitors	RSL3, ML162	GPX4 active site (selenocysteine)	Covalently bind and inactivate GPX4 → Failure to reduce lipid peroxides → Membrane damage	Pancreatic cancer, Lymphoma	[115]
Iron Metabolism Modulators	Ferric ammonium citrate (FAC), Deferoxamine	Iron ion homeostasis	FAC increases Fe^2+^ (promotes Fenton reaction); Deferoxamine chelates iron (modulates iron-dependent death)	Breast cancer, Lung cancer	[116,117]
Lipid Metabolism Modulators	A939572 (SCD1 inhibitor)	Stearoyl-CoA desaturase (*SCD1*)	Inhibit MUFA synthesis → Increased PUFA proportion in membrane phospholipids	Colorectal cancer, Glioma	[118]
FSP1 Inhibitors	iFSP1	Ferroptosis suppressor protein 1 (*FSP1*)	Block FSP1-CoQ10 pathway → Inhibit the antioxidant effect of ubiquinol (CoQ10H_2_)	Melanoma, Colon cancer	[119]
DHODH Inhibitors	BAY-2402234	Dihydroorotate dehydrogenase (*DHODH*)	Inhibit mitochondrial CoQ synthesis → Block compensatory antioxidant pathway	Acute myeloid leukemia (AML)	[120]

**Table 4:** Genes modulate cancer cell susceptibility to ferroptosis via distinct pathways.

Gene	Regulatory Mechanism	Effect on Ferroptosis	References
*p53*	Represses *SLC7A11* expression (reducing cystine uptake); Activates *SAT1*/*ALOX15*, *GLS2* (promoting lipid peroxidation and ROS generation)	Promotes (in specific contexts)	[125,126]
*SLC7A11*	Light chain subunit of system Xc^−^, mediates cystine uptake. High expression enhances GSH synthesis, inhibiting ferroptosis; Repression by *p53* or *NRF2* promotes ferroptosis	Inhibits	[113]
*SAT1*	*p53* target gene; Activates *ALOX15*-mediated lipid peroxidation; Disrupted polyamine metabolism exacerbates oxidative stress	Promotes	[127]
*FSP1*	Scavenges lipid radicals by reducing coenzyme Q10 (CoQ10), constituting a GPX4-independent antioxidant pathway	Inhibits	[128]
*ACSL4*	Catalyzes esterification of PUFAs into membrane phospholipids, providing substrates for lipid peroxidation. Its knockout significantly reduces ferroptosis sensitivity	Promotes	[129]
*NRF2*	Activates antioxidant genes (e.g., *SLC7A11*, *GCLC* [GSH synthesis enzyme]), suppressing ROS accumulation	Inhibits	[130]
*ALOX12/15*	Directly oxidizes PUFAs to generate lipid peroxides. *p53* upregulates *ALOX15* activity via *SAT1*	Promotes	[131]

## Data Availability

No new data were created or analyzed in this study. Data sharing is not applicable to this article.

## Abbreviations

The following abbreviations are used in this manuscript:

AA	Arachidonic acid
ABC	ATP-binding cassette
*ACSL4*	Acyl-CoA synthetase long-chain family member 4
AdA	Adrenic acid
ADCs	Antibody-drug conjugates
AI	Artificial intelligence
AP	Apurinic/apyrimidinic
BCRP	Breast cancer resistance protein
BER	Base excision repair
c-FLIP	Cellular FLICE-like inhibitory protein
*CHK*	Csk homologous kinase
CMS	Consensus molecular subtypes
CRC	Colorectal cancers
CSC	Cancer stem cell
CTC	Circulating tumor cell
ctDNA	Circulating tumor DNA
CYP	Cytochrome P450
DAMPs	Damage-associated molecular patterns
DCs	Dendritic cells
DFO	Deferoxamine
DMT1	Divalent metal transporter 1
DNMTi	DNA methyltransferase inhibitors
DNMTs	DNA methyltransferases
DOX	Doxorubicin
DSBs	Double-strand breaks
EMT	Epithelial–mesenchymal transition
EPR	Enhanced permeability and retention
ETC	Electron transport chain
*FASN*	Fatty acid synthase
FPN	Ferroportin
5-FU	5-fluorouracil
GCL	Glutamate-cysteine ligase
GPx	Glutathione peroxidase
*GPX4*	Glutathione peroxidase 4
GS	Glutathione synthetase
GSH	Glutathione
GSR	Glutathione reductase
GSSG	Oxidized glutathione
*GSTs*	Glutathione S-transferases
HCC	Hepatocellular carcinoma
HDACi	Histone deacetylase inhibitors
*HDACs*	Histone deacetylases
*HMTs*	Histone methyltransferases
HRD	Homologous recombination deficiency
HREs	Hypoxia response elements
HRR	Homologous recombination repair
*LCN2*	Lipocalin 2
LIP	Labile iron pool
LOOH	Lipid hydroperoxides
LOX	Lipoxygenase
*LPCAT3*	Lysophosphatidylcholine acyltransferase 3
MAPK	Mitogen-activated protein kinase
MDR	Multidrug resistance
MMR	Mismatch repair
MOMP	Mitochondrial outer membrane permeabilization
MRP1	Multidrug resistance-associated protein 1
MSI	Microsatellite instability
MUFAs	Monounsaturated fatty acids
NATs	N-acetyltransferases
NBDs	Nucleotide-binding domains
ncRNA	non-coding RNA
*Nrf2*	Nuclear factor erythroid 2-related factor 2
NSCLC	Non-small-cell lung cancer
OTICs	Ovarian tumor-initiating cells
*PARP*	Poly (ADP-ribose) polymerase
PCET	Proton-coupled electron transfer
PDX	Patient-derived xenograft
PE	Phosphatidylethanolamine
PEBP1	Phosphatidylethanolamine-binding protein 1
PEG	Polyethylene glycol
P-gp	P-glycoprotein
PLGA	Poly (lactic-co-glycolic acid)
*PTGS2*	Prostaglandin-endoperoxide synthase 2
PUFAs	Polyunsaturated fatty acids
PXR	Pregnane X receptor
ROS	Reactive oxygen species
SFAs	Saturated fatty acids
*SOD*	Superoxide dismutase
SQS	Squalene synthase
*SREBP1*	Sterol regulatory element-binding protein 1
SSBR	Single-strand break repair
SSBs	Single-strand breaks
SULTs	Sulfotransferases
TFR1	Transferrin receptor 1
*TFRC*	Transferrin receptor
TMDs	Transmembrane domains
TME	Tumor microenvironment
UGTs	UDP-glucuronosyltransferases
WES	Whole-exome sequencing
γ-GCS	γ-glutamylcysteine synthetase
